# Case report: *ALK*-rearranged spindle and epithelioid cell neoplasms with S100 and CD34 co-expression: Additional evidence of kinase fusion–positive soft tissue tumors

**DOI:** 10.3389/fonc.2022.1007296

**Published:** 2022-10-26

**Authors:** Shao-Jie Sheng, Ju-Ming Li, Qin-He Fan, Yang Liu, Shao-Yu Chen, Ming Zhao, Qi-Xing Gong

**Affiliations:** ^1^ Department of Pathology, The First Affiliated Hospital of Nanjing Medical University, Nanjing, China; ^2^ Department of Pathology, The First People’s Hospital of Changzhou, Changzhou, China; ^3^ Department of Orthopedics, The First Affiliated Hospital of Nanjing Medical University, Nanjing, China; ^4^ R & D department, Guangzhou LBP Medicine Science & Technology Co., Ltd, Guangzhou, China; ^5^ Cancer Center, Department of Pathology, Zhejiang Province People’s Hospital, People’s Hospital of Hangzhou Medical College, Hangzhou, China

**Keywords:** *ALK*, *PLEKHH2*, *EML4*, S100 and CD34 co-expression, soft tissue tumor

## Abstract

*ALK* rearrangements have rarely been reported in S100- and CD34-co-expressing soft tissue neoplasms with lipofibromatosis-like neural tumor (LPFNT) pattern or stromal and perivascular hyalinization, mimicking *NTRK*-rearranged spindle cell tumors. Here, we reported *ALK* fusions involving related partner genes in two adult soft tissue tumors with S100 and CD34 co-expression, and conducted a literature review of mesenchymal tumors harboring *ALK* or other kinase fusions. Case 1 was a 25-year-old female who underwent excision of a soft tissue mass in the anterior thigh region. Morphologically, the tumor was composed of spindle cells adjacent to epithelioid cells embedded in myxedematous and hyalinized stroma, with infiltrative boundary. Spindle cells mixed with inflammatory infiltration resembling inflammatory myofibroblastic tumor (IMT) were seen sporadically. However, brisk mitosis and focal necrosis was also observed, indicating an intermediate-grade sarcoma. In case 2, the left side of the neck of a 34-year-old man was affected. The tumor was composed of monomorphic spindle cells arranged in fascicular growth or patternless pattern, with stromal and perivascular hyalinization. Sparse inflammatory cell infiltration was also observed. Both tumors showed CD34, S100, and ALK-D5F3 immunoreactivity. Next generation sequencing (NGS) test identified a *PLEKHH2::ALK* fusion in case 1, which was confirmed by RT-PCR and Sanger sequencing, whereas the RT-PCR (ARMS method) test detected an *EML4::ALK* fusion in case 2. In conclusion, this study expands the morphological and genetic landscape of tumors with S100 and CD34 co-expression harboring kinase fusions, and suggests that kinase fusion–positive mesenchymal neoplasms are becoming an enlarging entity with a variety of morphological patterns.

## Introduction

According to the 2020 WHO classification of soft tissue tumors (STTs), *NTRK*-rearranged spindle cell tumors are an emerging entity, which spans a wide spectrum of morphologies and histologic grades, with frequent immunohistochemical co-expression of S100 and CD34. Notably, the family of this entity is expanding, as tumors with similar clinicopathological features and morphology but alternative kinase genes fusions are constantly identified; among them, STTs with *ALK* gene rearrangement have emerged as a recent hot spot (1–20).

The *ALK* gene (2p23) encodes a cell membrane receptor tyrosine kinase (RTK), which plays an important role in brain development and specific neurons in the nervous system. Oncogenic activation of ALK kinase following *ALK* rearrangement has been reported in a variety of tumors, including non-small cell lung cancer (NSCLC), anaplastic large cell lymphoma (ALCL), IMT, epithelioid fibrous histiocytoma (EFH) (21), *ALK*-positive histiocytosis (22), renal cell carcinoma (23), thyroid cancer (24), secretory carcinomas (25), and gastrointestinal stromal tumor (GIST) (26). Recently, *ALK* rearrangements have been reported in S100- and CD34-co-expressing soft tissue tumors (5–14). A provisionally termed entity, superficial *ALK*-rearranged myxoid spindle cell neoplasm, has been coined to emphasize the characteristic swirling pattern of spindle cells arranged in myxoid or myohyaline stroma (6). Later, Kao YC et al. reported an additional case of superficial *ALK*-rearranged spindle cell neoplasm, which showed ovoid tumor cells predominantly arranged in reticular and cord-like patterns in a hyalinized stroma, with only focal presence of whorl-like pattern (7). However, the emerging tumor was also characterized by frequent S100 protein and CD34 co-expression, perivascular hyalinization, and collagenous stroma, and it partly showed LPFNT pattern, which could not sufficiently distinguish it from other *ALK*-rearranged tumors with S100 and CD34 co-expression. Furthermore, infantile fibrosarcoma (IFS)-like pattern, which is normally reported in the wide morphological spectrum of *NTRK*-rearranged STTs, including infantile fibrosarcoma and *NTRK*-rearranged spindle cell tumors, has also been documented with *ALK* rearrangements (8, 27).

Therefore, more cases are needed to recognize the innate character of such soft tissue tumors with *ALK* rearrangements and improve their classification and nomenclature. In this study, we identified two S100- and CD34-co-expressing STTs with *ALK* rearrangement and summarized the clinicopathological characteristics of the reported kinase fusion–positive mesenchymal neoplasms, hoping to enlighten new ideas.

## Case presentation

### Clinicopathological findings

**Figure 1:**
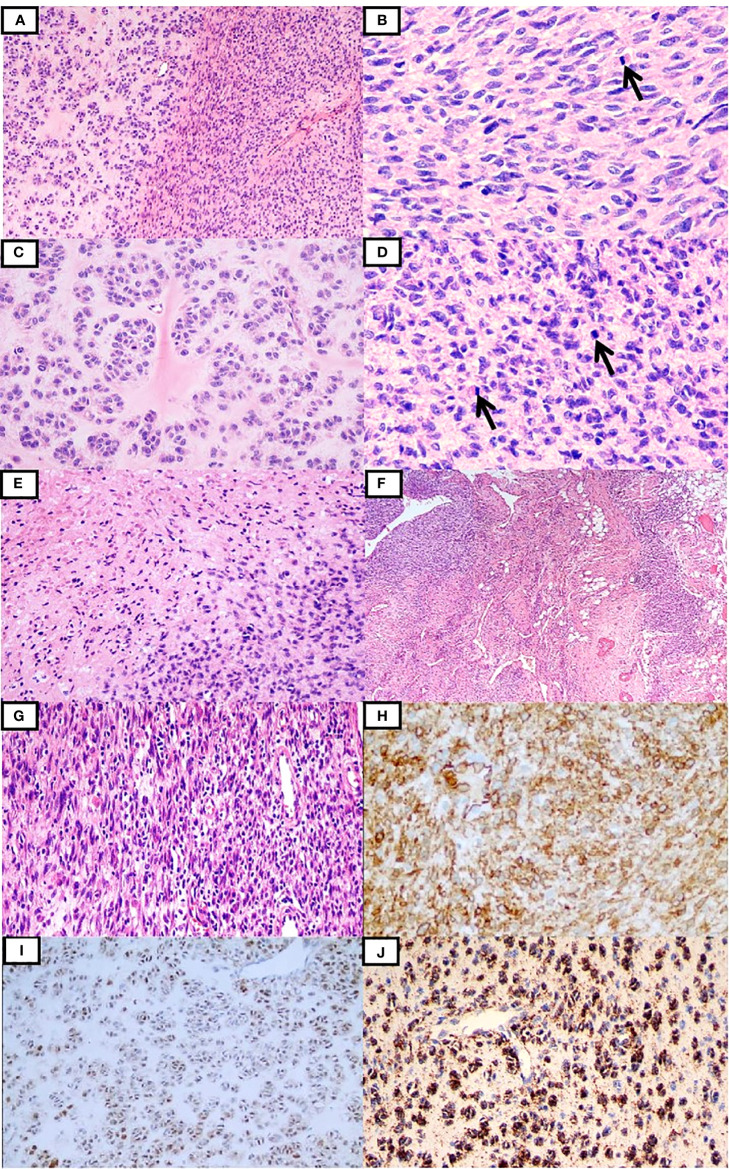
Clinicopathological findings of the tumor in case 1. The tumor was composed of the spindle and epithelioid cells embedded in myxedematous and hyalinized stroma **(A)**. The spindle cells were focally arranged in the intersecting fascicles with frequently observed mitotic figures **(B)**, while the epithelioid cells were arranged in the nest- or cord-like pattern in the myohyaline background **(C)**. In some areas, the tumor cells were more cellular with relatively brisk mitoses **(D)**. Focal necrosis was recognized **(E)**. Tumor cells infiltrating the surrounding adipose tissues and thin-walled branching vessels were seen **(F)**. Inflammatory cell infiltration was observed locally **(G)**. Tumor cells were positive for CD34 **(H)**, S100 **(I)**, and ALK-D5F3 **(J)**.

Case 1 was a 25-year-old woman with an egg-sized, movable, painless mass in the left anterior thigh region for more than 1 year, with a gradual increase in size associated with pain for 2 months. Magnetic resonance imaging (MRI) suggested an intramuscular mass between the anterior rectus and vastus lateralis muscles in the left thigh (**Supplementary Figure 1**). The patient underwent resection of the mass. Macroscopically, the resected specimen comprised a soft solid tumor mass measuring 6.5 × 3.5 × 2.8 cm with a gray-white, fleshy, or myxoid cut surface. Microscopically, the tumor was composed of spindle cells juxtaposed with epithelioid cells embedded in myxedematous and hyalinized stroma (**Figure 1A**), partially infiltrating surrounding striated muscles and adipose tissue. The spindle cells were arranged in sheet-like, intersecting fascicles, or in patternless patterns, showing indistinct cytoplasmic borders and moderate nuclear pleomorphism (**Figure 1B**). The epithelioid cells were arranged in a nest- or cord-like pattern in a myohyaline background with ample eosinophilic cytoplasm and round to ovoid nuclei (**Figure 1C**). The mitotic figures (MFs) were plentiful, especially in the cellular area (about 8 MFs/10 high-power fields (HPFs)) (**Figure 1D**). Focal hemorrhage and necrosis were also observed in the spindle cell area (**Figure 1E**). Prominent branching of thin-walled blood vessels of different sizes was also found (**Figure 1F**). At the periphery, some spindle tumor cells admixed with infiltrating inflammatory cells, closely resembling IMT (**Figure 1G**). According to the French Federation of Cancer Centers Sarcoma Group (FNCLCC) grading, the morphology of the neoplasm was intermediate grade. The tumor cells were immunohistochemically positive for CD34 (**Figure 1H**), S100 (**Figure 1I**), ALK-D5F3 (**Figure 1J**), H3K27me3, vimentin, and CD99 (paranuclear dot-like staining), and they were negative for STAT6, CK-pan, EMA, desmin, SMA, CD31, WT-1, and pan-TRK. The average Ki-67 index was 35%. The patient underwent postoperative radiotherapy(70Gy/35F), and there were no signs of recurrence or metastasis 48 months after surgery.

**Figure 2:**
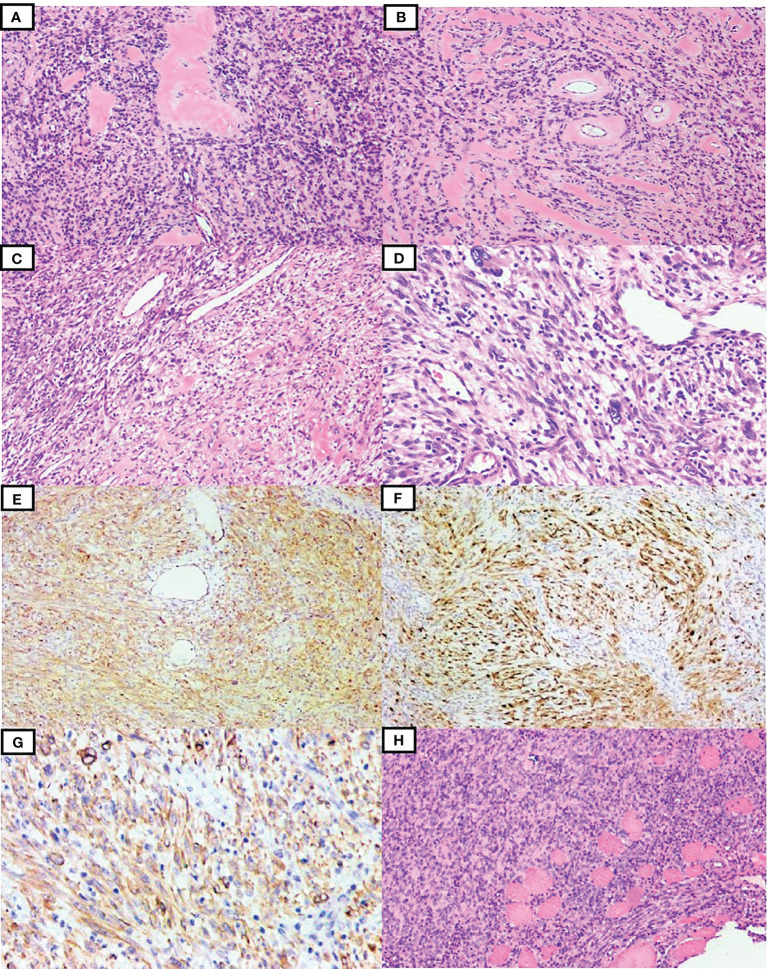
Clinicopathological findings of the tumor in case 2. The tumor consisted of spindle-shaped mesenchymal cells with stromal and perivascular hyalinization **(A, B)**. Focal staghorn vessels and clusters of clear cytoplasmic cells were observed **(C)**. Giant multinucleated tumor cells and inflammatory infiltration were also seen **(D)**. The tumor cells were diffusely positive for CD34 **(E)**, S100 **(F)**, and ALK-D5F3 **(G)**. The relapsed tumor showed diffuse proliferation of compact spindle cells, also infiltrating striated muscles **(H)**.

Case 2 was a 34-year-old man with a mass on the left side of his neck. The tumor was marginally removed without further treatment. Grossly, the mass was partially encapsulated measuring 8 × 5 × 4 cm in size. The texture was soft, and the cut surface was gray-white to gray-yellow. Microscopically, the lesion was composed of spindle-shaped mesenchymal cells infiltrating the fat tissue and striated muscle, with stromal and perivascular hyalinization (**Figures 2A, B**). A higher-power view showed bland spindle cells arranged in a patternless pattern with fusiform nuclei and fine chromatin. Focal clusters of cells showed clear cytoplasm (**Figure 2C**). Pleomorphic and multinucleate cells were occasionally seen. Sparse inflammatory cell infiltration was also observed (**Figure 2D**). The mitotic count was 1 MF/10 HPFs. Necrosis was not found. According to FNCLCC grading, the morphology of the neoplasm was low grade. The tumor cells were immunohistochemically positive for CD34 (**Figure 2E**), S100 (**Figure 2F**), and ALK-D5F3 (**Figure 2G**), and negative for AE1/3, SMA, desmin, STAT6, and SOX10. H3K27me3 staining was retained. The available clinical follow-up information of the patient revealed that the tumor recurred at the original site 27 months after surgery. Pathologically, the relapsed tumor showed similar morphology and immunophenotype to the original tumor, with more compact tumor cells (**Figure 2H**).

### Molecular findings

**Figure 3:**
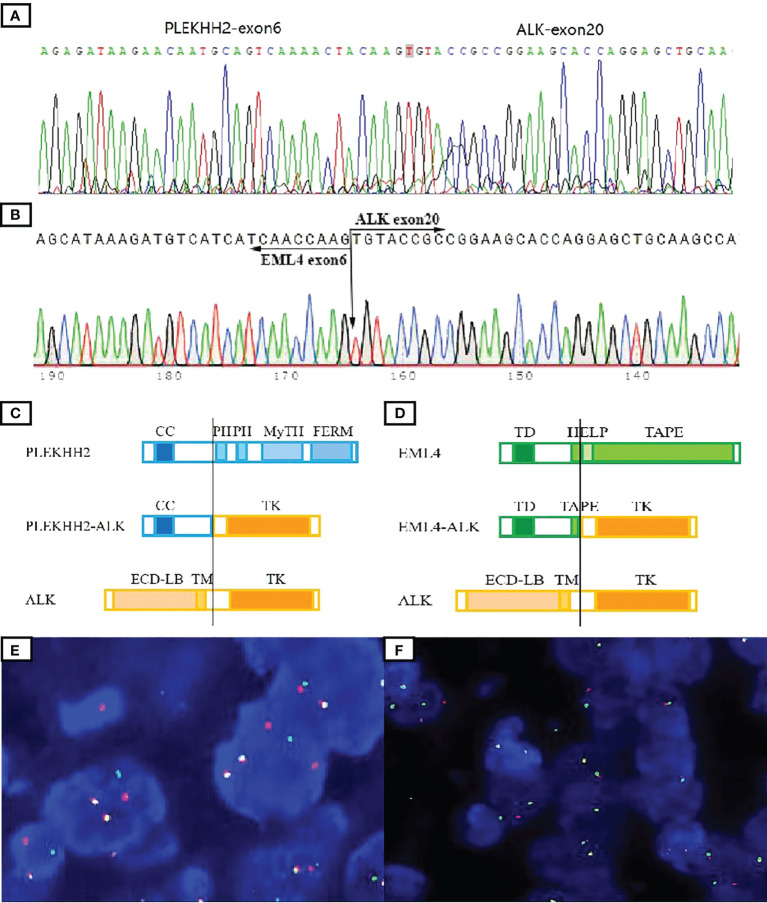
Molecular tests to validate ALK fusions. The presence of the *PLEKHH2::ALK* fusion transcript was validated by Sanger sequencing **(A)**. Direct sequencing of the purified RT-PCR products revealed the chimeric transcripts between exon 6 of *EML4* and exon 20 of *ALK* **(B)**. Schematic representation of the predicted chimeric proteins **(C, D)**. By ALK break-apart FISH test, most tumor cells in both cases demonstrated a signal pattern consisting of isolated 5′ (green) and isolated 3′ (orange), along with fused 3′/5′ signals **(E, F)**. TD, trimerization domain; HELP, hydrophobic motif in EML proteins; TAPE, tandem atypical propeller domain; ECD-LB, extracellular ligand-binding domain; TM, transmembrane domain; TK, tyrosine kinase domain; CC, a-helical coiled-coil domain; PH, Pleckstrin homology domain; MyTH, MyTH4 domain; FERM, four-point-one ezrin radxin mesoin family.

Genomic DNA was extracted from formaldehyde-fixed paraffin-embedded (FFPE) tumor tissues using the QIAamp DNA mini kit (Qiagen, Hilden, Germany). Targeted deep sequencing of mutational hot spots was conducted using a capture-based targeted sequencing panel (Burning Rock Biotech, Guangzhou, China), including a panel of 520 genes to detect genomic alterations including single base substitution, short and long insertions/deletions, copy number variations, gene fusions, and rearrangement. NGS test identified a transcript comprising intron 6 of *PLEKHH2* and intron 20 of *ALK* in case 1, which was validated by RT-PCR and Sanger sequencing (**Figure 3A**).

Genomic RNA was extracted from tumor FFPE tissues using RNeasy FFPE (Qiagen, Hilden, Germany) and reverse transcribed using SuperScript IV First-Strand Synthesis System (Invitrogen, Carlsbad, CA, USA). The mutation of *EML4::ALK* was detected according to the ARMS methods using a human multigene mutation detection kit (PCR fluorescence probe method) (Amoy Diagnostics Co. Ltd., Xiamen, China). The PCR product was analyzed by Sanger sequencing using Big Dye Terminator Sequencing kit (Applied Biosystems, Foster City, CA, USA). ARMS test detected a transcript comprising exon 6 of *EML4* and exon 20 of *ALK* in case 2 (**Figure 3B**).

The predicted chimeric proteins consisted of an N-terminal part with the coiled-coil domains of *PLEKHH2* or *EML4* and a C-terminal part with the complete kinase domain of *ALK* (**Figures 3C, D**).

Fluorescence *in situ* hybridization (FISH) analysis was performed on 3-µm-thick FFPE tumor sections using the dual-color break-apart probe of *ALK* (Abbot Molecular, Abbott Park, IL, USA). A hundred nonoverlapping cells were scored, and more than 20% of tumor cells with abnormal signals were considered positive for gene rearrangement. FISH results confirm *ALK* rearrangements in both cases (**Figures 3E, F**).

## Discussion

In this study, we reported two cases of STTs with S100 and CD34 co-expression harboring ALK gene rearrangements with some distinct features. Morphologically, case 1 was intermediate-grade sarcoma composed of uniform spindle cells and epithelioid cells arranged in myxedematous and hyalinized stroma, with brisk mitosis, focal necrosis, and inflammatory cell infiltration. Although most of the kinase fusion–positive STTs were defined as spindle cell tumors, epithelioid cells have been observed in some areas of S100- and CD34-co-expressing tumors harboring *RAF1*, *BRAF*, and *ALK* gene rearrangements (3, 9, 19). Myxedematous stroma has been found in some cases of *ALK-*rearranged STTs with S100 and CD34 co-expression (6–11). However, tumor cells with brisk mitosis and focal necrosis, which were the features of intermediate- to high-grade sarcoma, have rarely been reported in *ALK-*rearranged STTs. The tumor in case 2 showed moderate to high cellular proliferation and stromal and perivascular hyalinization, which are consistent with morphological features reported by Suurmeijer et al. (15). Similar to other reported S100- and CD34-co-expressing mesenchymal tumors harboring *ALK* rearrangement (6, 8, 9, 12, 13), inflammatory infiltration was found in both our cases. However, case 1 even showed IMT-like morphology, suggesting IMT in the differential diagnosis, but then we discarded the hypothesis due to the absence of myogenic expression and S100 and CD34 co-expression. To the best of our knowledge, IMT-like morphology has not been revealed in S100- and CD34-co-expressing mesenchymal tumors harboring *ALK* rearrangement. Nevertheless, it has been reported in *NTRK*-rearranged spindle cell tumors, presented primarily (28) or as a morphological transformation after chemotherapy (29). Based on the case reported here and the literature reviewed in the Introduction section, we speculate that similar to *NTRK*-rearranged spindle cell tumors, *ALK*-rearranged soft tissue tumors also span a wide spectrum of morphologies and histologic grades. Furthermore, the IMT-like pattern, analogous to the LPFNT pattern, might overlap with other patterns in the wide spectrum of kinase fusion–positive mesenchymal neoplasms.

Genetically, the tumor in case 1 was identified to harbor *PLEKHH2::ALK* fusion gene, whereas the tumor in case 2 showed *EML4::ALK* gene fusion. The *PLEKHH2* gene (2p21) encodes an intracellular protein highly enriched in renal glomerular podocytes, which plays a structural and functional role in the podocyte foot processes. The presence of a putative a-helical coiled-coil domain was observed in the N-terminus of PLEKHH2 (30). The *EML4* gene (2p21) encodes a microtubule-associated protein with a coiled-coil domain and may generate abnormal fusion with *ALK*, which has been identified in lung adenocarcinoma, breast cancer, colorectal cancer, IMT, and S100- and CD34-co-expressing neoplasms. Commonly, *ALK* fusions could activate the ALK kinase domain without a ligand through autophosphorylation due to dimerization. Both of the fusion genes in our study contained the entire intracellular kinase domain of *ALK* and the coiled-coil domain of the fusion partner genes, which mediated dimerization and activation of the ALK kinase domain. Therefore, the fusion proteins were presumed to have an oncogenic function.

Recently, an emerging class of spindle cell tumors characterized by frequent S100 protein and/or CD34 co-expression and recurrent tyrosine kinase fusions, including *BRAF*, *RAF1, RET, MET*, *ROS1*, and *ALK*, has been documented, although it is unclear whether these tumors should be classified into one category (31). We tried to summarize their features and find some commonalities listed hereafter. First, the related kinase fusion genes are predominantly tyrosine kinase genes, which regulate downstream signaling pathways, including the MAPK/ERK, PI3K/AKT, and JAK3-STAT3*. BRAF* and *RAF1* even constitute the MAPK pathway components. Second, most of the oncogenic activation of kinase genes is through rearrangement. The kinase domain is reserved, and the partner gene is responsible for dimerization or other ways to mediate the activation of the kinase domain. Third, kinase fusion–positive neoplasms have been proven to be effective for targeted therapy (13, 27), not only in mesenchymal tumors but also in various epithelial neoplasms. Finally, these kinase fusion–positive mesenchymal neoplasms share similar clinicopathological features with *NTRK*-rearranged spindle cell tumors.

We have generalized the features of 47 mesenchymal neoplasms with oncogenic kinase alterations akin to *NTRK*-rearranged mesenchymal neoplasms searched in the available published literature, including 10 cases positive for RAF1, eight for RET, four for BRAF, 21 for *ALK*, one for *MET*, one for *ROS1*, and two for *ABL1* gene rearrangements (1–20). Among them, 15 were found in children (<10 years), seven were found in adolescents (age range of 10–20 years), and 25 were found in adult patients (>20 years old). Both sexes were affected (27 females and 20 males). The tumors were most commonly located in soft tissues of the trunk and extremities, while a few occurred in the head and neck region, viscera, and even skeleton. Tumor size ranged from 0.5 cm to 14 cm in 29 tumors with available data. The 47 tumors spanned a wide spectrum of morphologies and histologic grades, showing monomorphic spindle cell proliferation in a haphazard arrangement with occasional components of epithelioid or pleomorphic cells. In addition to the unified features mentioned above, some cases seem to show overlapped characteristics. Myxoid stroma was observed in some cases (14/47) and seemed to be more frequently present in *ALK*-rearranged tumors (6–11, 16). Some *ALK*-, *RAF1*-, or *RET*-rearranged tumors were characterized by the presence of tumor cells arranged in concentric whorls, which was also observed in *NTRK*-rearranged tumors (6, 7, 17, 18, 32). Staghorn or hemangiopericytoma-like vessels were also observed in some *ALK*-, *RET*-, *RAF1*-, or *BRAF*-rearranged tumors (8/47), which has also been recognized as one of the characteristics of *NTRK*-rearranged STTs (6, 14, 16, 18, 19). Inflammatory infiltration was readily witnessed in nearly one-third of cases, closely correlating with LPFNT morphology (20). Tumors with low-grade morphological features were common (32/47, 68.1%), while intermediate- to advanced-grade tumors were relatively rare. Certainly, with the deepening understanding of these tumors, some less common features will be reported and summarized, even under the name of other provisionally termed entities. The prognosis of the tumors appears to be related to histologic grade. Low-grade tumors with positive margins showed a propensity for local recurrence, whereas high-grade tumors showed aggressive clinical behavior and metastasized to lungs or other organs. Thus, in view of increasing cases of kinase fusion–positive mesenchymal neoplasms, we believe that the emerging entity of mesenchymal neoplasms with oncogenic kinase alterations akin to NTRK-rearranged spindle cell tumors could develop into a constantly expanding family of kinase fusion–positive soft tissue tumors.

## Conclusions

Herein, we reported two spindle and epithelioid cell neoplasms with S100 and CD34 co-expression showing recurrent *ALK* rearrangements. Our report adds to the morphological and genetic spectrum of the novel, recently described entity with S100 and CD34 co-expression harboring kinase fusions. We believe that more reported cases will unveil the panoramic view of the clinicopathological features of kinase fusion–positive STTs and improve patient treatment strategies and prognosis *via* targeted therapies.

## Data availability statement

The original contributions presented in the study are included in the article/**Supplementary Material**. Further inquiries can be directed to the corresponding authors.

## Ethics statement

The studies involving human participants were reviewed and approved by the Ethics Committee of the First Affiliated Hospital of Nanjing Medical University. The patients/participants provided their written informed consent to participate in this study.

## Author contributions

S-JS, J-ML, Q-HF, MZ, and Q-XG designed the study. S-JS, J-ML and YL participated in patient treatment and analyzed clinical data. S-YC performed molecular testing and analyzed the data. S-JS and J-ML drafted the manuscript. Q-XG and MZ supervised the work and revised the manuscript. Q-HF helped revised the manuscript. All authors contributed to the article and approved the submitted version.

## Funding

This work was supported by Technology Support Programs of Suqian city (S201519), Natural Science Foundation of Zhejiang Province (LY21H160052), and Zhejiang Provincial Medicine and Health Research Foundation (2023KY040). The funders did not have any role in the design and conduct of the study, the analysis and interpretation of the data, and preparation of the manuscript.

## Conflict of interest

Author S-YC was employed by Guangzhou LBP Medicine Science & Technology Co., Ltd.

The remaining authors declare that the research was conducted in the absence of any commercial or financial relationships that could be construed as a potential conflict of interest.

## Publisher’s note

All claims expressed in this article are solely those of the authors and do not necessarily represent those of their affiliated organizations, or those of the publisher, the editors and the reviewers. Any product that may be evaluated in this article, or claim that may be made by its manufacturer, is not guaranteed or endorsed by the publisher.

## Supplementary material

Supplementary Figure 1 Imaging studies of the left thigh in case 1. The lesion situated between the anterior rectus and vastus lateralis muscles **(A)** and was slightly hypointense in T1 weighted imaging **(B)** and significant hyperintense in T2 weighted imaging **(C)**.
