# Supplementary figures and images for: Case report: ALK-rearranged spindle and epithelioid cell neoplasms with S100 and CD34 co-expression: Additional evidence of kinase fusion–positive soft tissue tumors
Source: Front Oncol. 2022 Oct 26;12:1007296. doi: 10.3389/fonc.2022.1007296 (PMC9643764; doi:10.3389/fonc.2022.1007296)

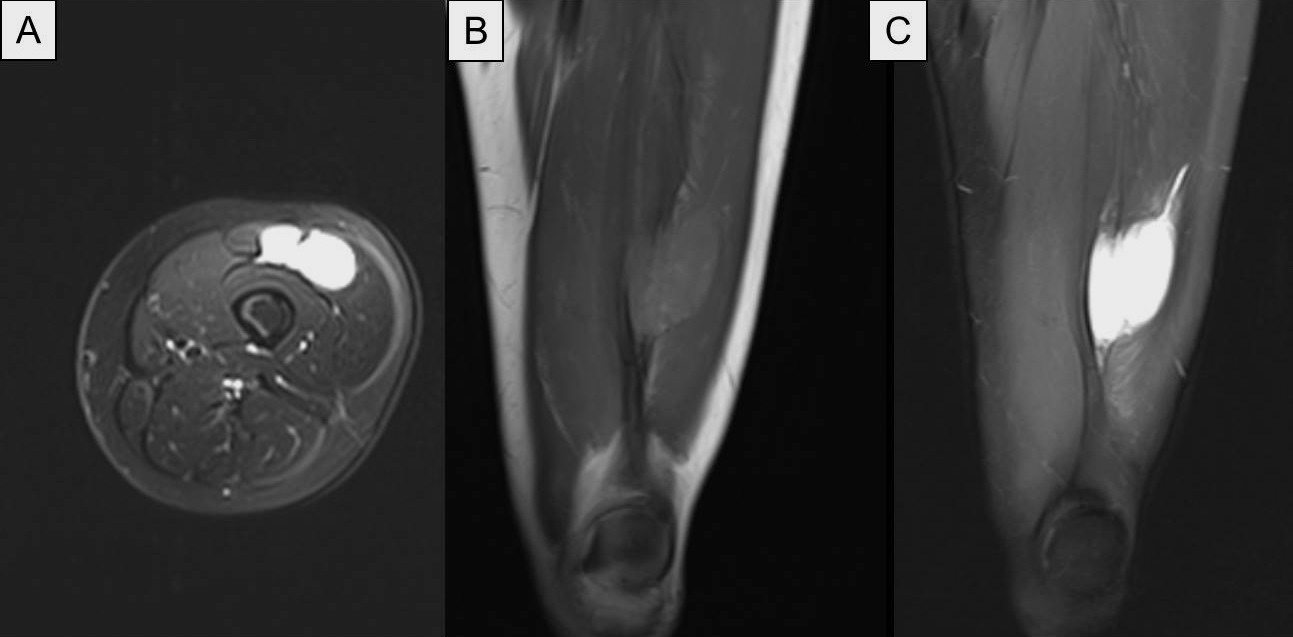

Supplement: Supplementary Figure 1 — Imaging studies of the left thigh in case 1. The lesion situated between the anterior rectus and vastus lateralis muscles (A) and was slightly hypointense in T1 weighted imaging (B) and significant hyperintense in T2 weighted imaging (C). [file Image_1.jpeg]
